# Numerical and experimental study on monitoring coal cracks with PZT sensor

**DOI:** 10.1038/s41598-023-28199-7

**Published:** 2023-01-18

**Authors:** Chengyao Zhu, Runzhi Li, Ke Gao, Zhiqiang Tang, Zeyi Liu, Lianzeng Shi

**Affiliations:** 1 College of Safety Science and Engineering, Liaoning Technical University, Huludao, 125105 Liaoning China; 2 College of Safety and Environmental Engineering, Shandong University of Science and Technology, Qingdao, 266590 China; 3 Key Laboratory of Mine Thermo-Motive Disaster and Prevention, Ministry of Education, Huludao, 125105 Liaoning China

**Keywords:** Electrical and electronic engineering, Coal

## Abstract

The rupture of coal pillar can lead to spontaneous combustion or collapse of goaf, which endangers the safety of workers. To explore the relationship between the crack depth of the coal structure and the signal received by the piezoelectric ceramic sensor, the output data of coal samples were analyzed by using the piezoelectric effect, combined with the experiment and ABAQUS simulation. Based on the signal amplitude, the output signal characteristics of the coal model with different crack depths were analyzed, and the evaluation index of coal crack cracking degree (*D*_*c*_) was defined. The results show that the piezoelectric fluctuation method can effectively identify the local cracks of coal. When the distance between the lead Piezoelectric Transducer (PZT) patch and crack position is constant, the amplitude of the PZT patch output signal will decay with the deepening of the crack depth, while the value of increases with the increase of crack depth. This study provides a theoretical basis for mine disaster prevention and control.

## Introduction

Coal spontaneous combustion is still one of the main causes of coal mine accidents, which can cause casualties and a serious waste of coal resources. Coal-seam's spontaneous combustion fire is mainly concentrated in the hidden space of the underground, such as the crushed stoop in the goaf, near the connection roadway, the stopping line, and the fall of ground and float coal accumulation of coal roadway^1^. Coal pillar in the mine mainly plays the role of isolation and protection support, In the process of coal cutting and tunnelling, different stress states and stress degrees will cause different forms of coal failure. When there is a crack in the coal body, it is difficult to observe by naked eye, and no abnormal occurrence. When the crack is further developed, there will be water leakage, air leakage and other phenomena, leading to the failure of isolated coal pillar, groundwater into the roadway, air into the goaf, causing floods, goaf spontaneous combustion and other accidents; The coal pillar that acts as the support protection may fracture after the crack, eventually leading to the collapse of the roadway and the ground collapse. Cracks can be detected at the early stage of cracks development, and effective prevention and control measures can be made in time to prevent further cracks development and prevent disasters. Due to the underground conditions and the particular situation of the coal pillar, it is difficult to quickly and accurately determine the location and range of the hidden fire source^2^. Therefore, it is necessary to develop an accurate, rapid, and real-time monitoring method for coal cracks^3,4^. The proposed method can improve reliability and reduce the structure's maintenance and detection costs. Previous studies focused on the evolution of the damage mechanism of as-mined coal structures from multiple perspectives, lacking the real-time monitoring of coal cracks. Owing to the defects of as-mined coal structures and the special environment underground, the safety evaluation of coal structures becomes more complicated.

In the coal mining process, the instability of coal is a prerequisite for the dynamic disaster of coal and rock, and the crack of coal is an important index to reflect the current coal situation. Therefore, monitoring coal cracks plays a significant role in coal mine safety^5–7^. In the 1920s, the British engineer Griffith proposed the Griffith theory, which can be applied to rock mechanics. That is, cracks in the object lead to changes in the stress state inside the object, resulting in crack expansion, connection, and penetration, thus leading to material destruction. Li et al. proposed a method to predict coal seam fractures using multi-component seismic data. To improve the resolution of anisotropy parameters in thin coal bed, they used the joint inversion of longitudinal wave (PP-wave) and fast longitudinal wave (PS1-wave), as well as split converted wave (PS-wave) horizontal tracking based on the pre-stack migrated real sections^8^. Nemat-Nasser et al. made some test specimens with single or multiple cracks for study, and they analyzed the process of the generation, expansion, and penetration of cracks. The relevant mechanical models were established based on the experimental results and elastic fracture mechanics theory. And the crack growth and rock splitting phenomenon in the presence of cracks were explained^9,10^. On this basis, Zhang et al. conducted an acoustic emission (AE) response experiment on coal samples under cyclic loading^11^. The results showed a positive correlation between AE parameters and stress. Under the cyclic loading condition, the coal sample's AE signal showed an obvious Kaiser effect. To further improve the early warning speed and accuracy of coal disasters, Liu et al. collected acoustic emission signals of coal samples in a uniaxial compression test (UCT), and the energy distribution and fractal characteristics of sound wave emission were studied^12^. They discovered that with the increase of load intensity, the AE waveform spectrum showed an inverted V-type trend, which indicated that they first increased and then decreased. Its law and characteristics were related to the material, deformation form (rupture and friction), and crack scale of coal. At the same time, Kong et al. constructed a uniaxial compressive load experimental system to conduct experimental studies on coal samples containing original cracks and discussed the dynamic evolution of increased load^13^. They pointed out that the cracks will be compacted during the compaction phase, producing only a small number of acoustic events. With the increase of stress, the original crack will expand into the yield stage of the coal sample, and more AE signals will be generated. Therefore, AE counts and cumulative counts can reflect the loading stage and cracks evolution process. In the same way, Dong et al. investigated the qualitative relationship between rock instability precursors and principal stress direction through rock acoustic emission experiments^14^. It provides a new analysis method for stability monitoring in practical engineering. In addition to studying acoustic emission characteristics and damage evolution of coal under compressive load, Sun et al. combined digital image processing technology to extract the crack parameters of the image. In addition, a geometric model was established according to the relationship between mining face, transportation roadway, and return air roadway. The occurrence of fractures was calculated, and a fast statistical method for coal seam fractures was proposed^15^. Cao et al. proposed a modified active contour without edges (C-V) model based on image enhancement techniques and obtained comprehensive crack information from coal and rock images^16^. Because the tomographic results are affected by many factors in practical small-scale applications, Dong et al. adopted a modified three-dimensional (3D) tomographic method that combines passive acoustic emission acquisition and active ultrasonic measurements^17^. However, this method does not apply to the case of significant differences in wave velocity within structures. It is more reliable and stable when the wave velocity changes slowly. Traditional geophysical exploration methods require a lot of preliminary investigation, which is greatly affected by various parameters and limited by wave velocity difference. For this reason, Dong et al. proposed a new method for empty region identification in the 2D complex structure, which laid a foundation for 3D space detection^18^. To meet the requirements of high-accuracy locating of complex three-dimensional hole-containing structures, Dong et al. Proposed a velocity-free Microseismic/acoustic emission (MS/AE) source location method^19^. This method can also be applied to other fields, such as non-destructive testing of acoustic emission positioning. To expand the comprehensive analysis method of charge induction, Ding et al. carried out the monitoring test of charge monitoring in the coal failure process by combining physical test and theoretical analysis. They obtained the law of charge time–frequency domain signal of coal different failure processes^20^. To develop and improve the monitoring technology and theory of charge induced by the failure of coal and rock, Lv et al. based on a uniaxial compression test, the damage statistical relationship between the loaded coal and induced charge signals was established and developed coal and rock charge monitoring device of induced charge suitable for complex conditions^21^. In the previous studies, the damage mechanism of raw coal structure was studied from many angles, and the real-time monitoring of coal cracks was lacking.

During the outline of the 13th 5-year plan for national economic and social development of the People's Republic of China, the critical technology research on risk identification, monitoring, and early warning and equipment research and development of coal mine typical coal rock power disasters were focused on. It is urgent to adopt scientific and technical means and methods to intelligently identify the damage degree to coal and rock structures. The emergence of intelligent materials provides an effective way for engineering structure health monitoring technology. Among many intelligent materials, piezoelectric ceramics, as the main representative, have made significant progress in the health monitoring and damage assessment of various metal, concrete, and other structures^22^. Kawiecki et al. measured the damage degree of the concrete beam by pasting Piezoelectric Transducer (PZT) sheets on both ends of the measured concrete beam^23^. The damage degree and location of the concrete beam are analyzed through the electrical signal of the PZT sheet at the acquisition end. The test results show that the PZT sheet can measure the damage to the concrete beam very well. Subsequently, to prove that the fluctuation analysis method can identify the generation and degree of structural damage. Seth et al. used Lamb waves to monitor different defect forms, such as composite material delamination, transverse layer cracks, and through-hole. They summarized the influence of actuator sensor position on the test and the excitation methods of various signals^24^. Then, Sun et al. pasted a PZT patch on the surface of concrete beams to excite stress waves and obtained the relationship between the wave peak value and wave velocity with stress^25^. The results show that this method can monitor the generation and propagation of cracks in concrete structures in real-time. To facilitate the placement of sensors and improve their available rate, Song et al. encapsulated piezoelectric ceramic sheets in the middle of two marble aggregates to form a "piezoelectric intelligent aggregate" and then buried the aggregate in the monitored structure to realize the effective combination between the two.

Moreover, the feasibility and effectiveness of this encapsulation form were confirmed by experiments. Song was the first scholar to use the piezoelectric active sensing method to research concrete crack damage monitoring in the world^26–28^. By pre-embedding piezoelectric intelligent aggregate, Sun et al*.* conducted a detailed study on the propagation characteristics of stress wave and sound field generated by PZT patch as a sound source in concrete medium and analyzed that the monitoring data collected by sensors could effectively reflect the existing damage and the development trend of structural health^29^. Hughi et al*.* used piezo sensors embedded in reinforced concrete structures as part of an active monitoring system and used the data collected to estimate crack width to propose a method for measuring crack width and locating crack locations^30^. Meanwhile, Markovic et al. used the finite element software ABAQUS as a platform to establish a numerical model of smart aggregate and analyze the wave propagation process in the model. The simulation constructed a damage monitoring system based on the piezo-sensors active sensing method. The propagation characteristics of stress waves under the influence of crack depth and hole diameter of concrete beam and the difference between them were studied. However, in this model, severe reflection occurs when the wave propagates to the boundary, and the coupling effect between the piezo-sensors and the concrete is not apparent^31^. In addition, piezoelectric sensors have a wide range of applications. Han et al. used active sensing methods to study the damage detection of four standard timber connections^32^.

The monitoring method based on piezo sensors still lacks real-time monitoring and identification of coal structure damage. Based on this, combined with numerical simulation and experimental demonstration, this paper realized the excitation and reception of stress wave in coal medium through the piezoelectric effect of PZT patch^33,34^. The potential-time curve will be obtained and normalized processing. The voltage amplitude and propagation time of stress wave propagating under different crack depth of the sample were analyzed, so as to effectively identify the cracking degree of coal. It provides a simple, economical, and reliable way to realize the online monitoring of coal structure.

## Method

### Design of experimental

#### Experimental instruments and materials

**Figure 1:**
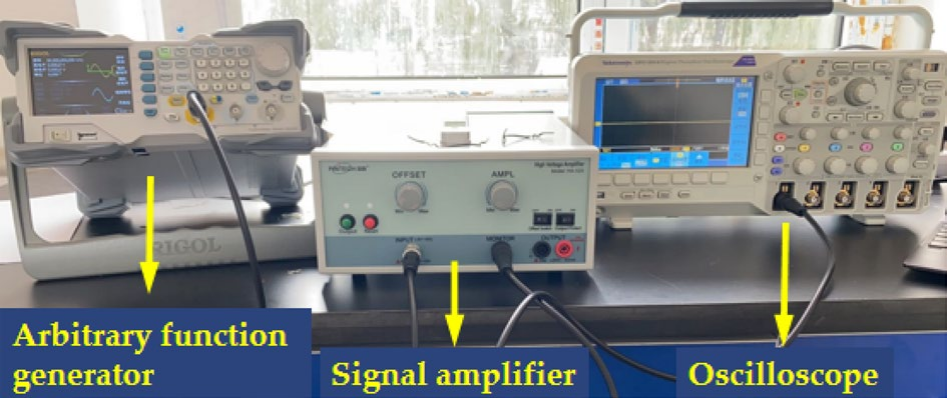
Coal crack monitoring system based on PZT sensor.

**Figure 2:**
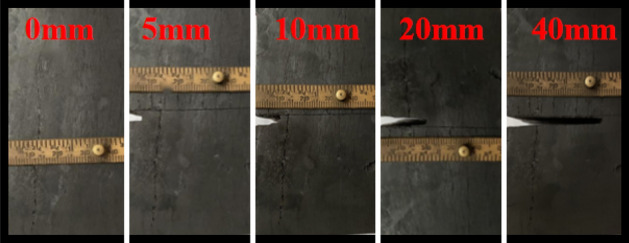
The coal samples tested.

As shown in Fig. 1, the experimental equipment includes: arbitrary function generator (RIGOL DG1062Z), signal amplifier (PINTECH HA-520), oscilloscope (Tektronix MDO3102), PZT patch and the sample of coal with size of 100 mm × 50 mm × 50 mm. As shown in Fig. 2, the width of the crack was about 2 mm in the middle of the sample, and the depth were 0 mm, 5 mm, 10 mm, 20 mm and 40 mm respectively.

#### Process of experimental

**Figure 3:**
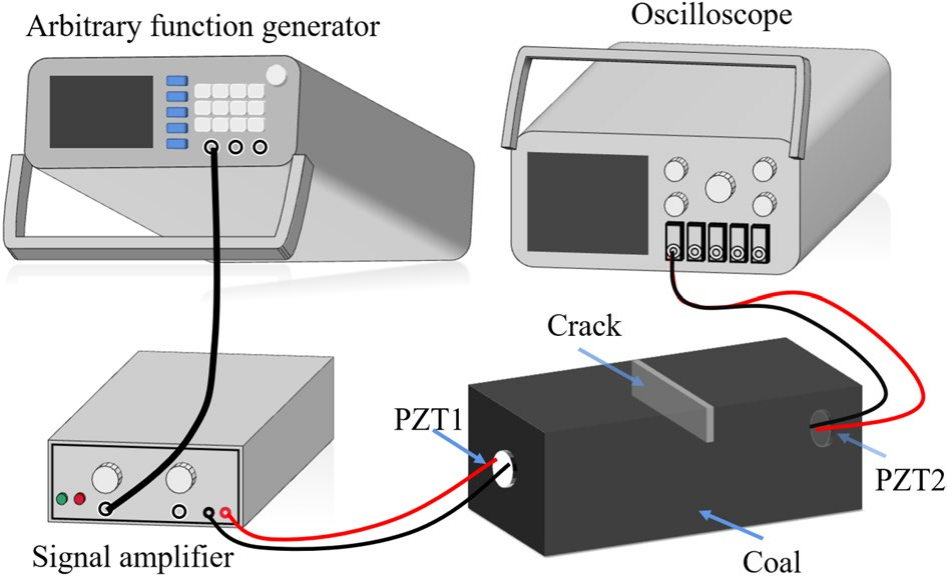
Experimental process diagram.

This paper used the principle of the fluctuation analysis method to attach the PZT patch to both ends of the coal surface. One end was used as a driver to transmit electrical signals, and the other was used as a sensor to receive signals. The arbitrary function generator is used to generate a specific voltage signal. Since the attenuation of stress wave in the specimen is very rapid, in order to ensure that the sensor at the receiving end can receive the transmitted signal completely, the signal will be amplified by signal amplifier. Then, the signal reaches the PZT 1. Under the inverse piezoelectric effect of the piezoelectric ceramic, the actuator at the incentive terminal caused the deformation of the surrounding medium to generate stress waves and propagated in the specimen. The final stress wave arrives in the PZT 2, causing the sensor deformation. Under the action of positive piezoelectric effect, the electric signal is generated. The electric signal will be displayed in the digital oscilloscope and the monitoring data will be extracted. The experimental procedure is shown in Fig. 3.

### Desgin of numerical simulation

ABAQUS finite element software has a perfect model building system. It can accurately analyze and calculate, realize the coupling of multiple physical fields of the model, and solve complex nonlinear problems, so it is selected as the numerical software for this study.

#### General equation

From the perspective of electromechanical coupling, the constitutive equation of piezoelectric materials is divided into four forms, each form corresponds to a material parameter. The equation is as follows (The first kind of equation is used in this paper):

Equation of the first kind (Electrical open-circuit *E* = 0, mechanical free *T* = 0)

1 $$\left\{ {\begin{array}{*{20}c} {S = s^{E} T + d^{t} E} \\ {D = dT + \varepsilon^{T} E} \\ \end{array} } \right.$$

where *S*, *D* and *T* are deformation, electric displacement and stress respectively; *s*^*E*^ is the short-circuit elastic compliance constant matrix; ${\varepsilon }^{T}$ is the free dielectric constant matrix; *d* is the piezoelectric strain constant matrix; *d*^*t*^ is the transpose matrix of *d*.

Equation of the second kind (Electrical open-circuit *E* = 0, mechanical clamping *S* = 0)

2 $$\begin{array}{*{20}c} {T = c^{E} S - e_{t} E} \\ {D = eS + \varepsilon^{S} E} \\ \end{array}$$

where, *c*^*E*^ is the short-circuit elastic stiffness constant matrix, *ε*^*S*^ is the clamping dielectric constant matrix, *e* is the piezoelectric stress constant matrix, and *e*_*t*_ is the transpose matrix of *e*.

Equation of the third kind (Electrical short-circuit *D* = 0, mechanical free *T* = 0)

3 $$\left\{ {\begin{array}{*{20}c} {S = s^{D} T + g_{t} D} \\ {D = - gT + \beta^{T} D} \\ \end{array} } \right.$$

where, *s*^*D*^ is the open-circuit elastic compliance constant matrix, ${\beta }^{T}$ is the free dielectric isolation rate matrix, *g* is the piezoelectric voltage constant matrix and *g*_*t*_ is the transpose matrix of *g*.

Equation of the third kind (Electrical short-circuit *D* = 0, mechanical clamping *S* = 0)

4 $$\left\{ {\begin{array}{*{20}c} {T = c^{D} S - h^{t} D} \\ {D = - hS + \beta^{S} D} \\ \end{array} } \right.$$

where, *c*^*D*^ is the short-circuit elastic stiffness constant matrix, ${\beta }^{S}$ is the clamping dielectric isolation matrix, *h* is the piezoelectric stiffness constant matrix *h*^*t*^ is the transpose matrix of *h*.

#### Physical model

**Figure 4:**
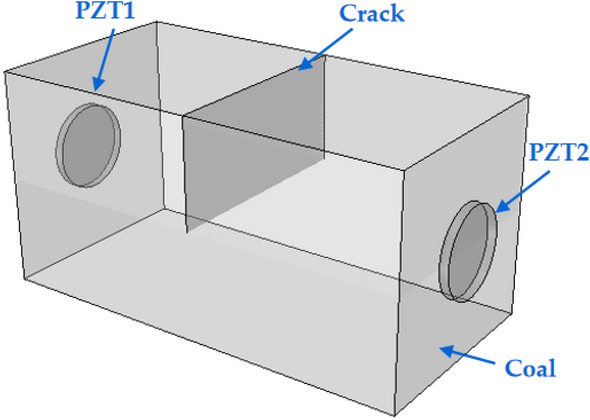
Simulation model of coal crack monitored by PZT.

A three-dimensional finite element numerical model was constructed, and the PZT patch was mounted at a preset position on the surface of the coal structure. The model was composed of three components: coal specimen, piezoelectric actuator, and piezoelectric sensor. The model dimension is shown in Table 1. PZT1 acted as an actuator to generate stress waves based on the piezoelectric effect of piezoelectric materials, and PZT2 served as a sensor to receive stress waves. The vertical crack depth of the model was set as 0 mm, 5 mm, 10 mm, 15 mm, 20 mm, 25 mm, 30 mm, 35 mm, and 40 mm, which was in the middle of the horizontal model. After the assembly was completed, the coupling connection between the PZT patch and the coal structure was realized by creating constraints to simulate the real working state. The finite element numerical model is shown in Fig. 4.

**Table 1:** The parts dimensions of the model.

Parts	Dimensions
Coal specimen	100 mm × 50 mm × 50 mm
PZT patch	Φ 20 mm × 2 mm

#### Selection of material parameters and element types

**Figure 5:**
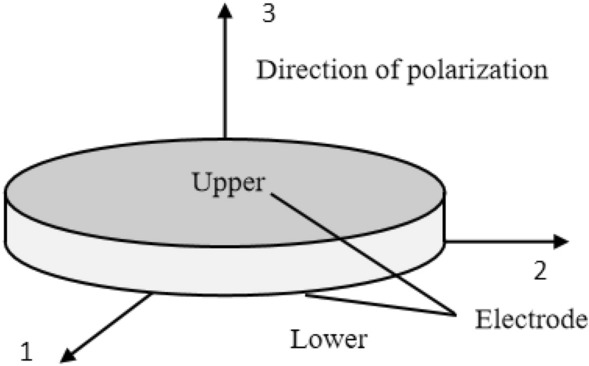
The polarization direction of the PZT patch.

It is noteworthy that the property of the piezoelectric ceramic material is anisotropic. Hence, the polarization direction of the piezoelectric ceramic material needs to be specified after setting its material parameters. The polarization direction of the PZT patch is shown in Fig. 5. In this simulation, the density, elastic matrix, dielectric constant matrix, and piezoelectric constant matrix of the PZT patch were defined by referring to the parameter setting of Chen^35^. The detailed parameters are shown in Table 2.

**Table 2:** PZT patch material parameter.

Material	Density ρ (kg/m^3^)	Dielectric constant (F/m)	Elastic constants (N/m^2^)	Piezoelectric constant (C/m^2^)
PZT	7450	D_11_ = 1.7 × 10^–8^	D_1111_ = 12.70 × 10^–10^	e_113_ = 17.03
			D_1122_ = 8.02 × 10^–10^	e_212_ = 17.03
			D_2222_ = 12.70 × 10^–10^	e_311_ = − 6.62
		D_22_ = 1.7 × 10^–8^	D_1133_ = 8.47 × 10^–10^	e_332_ = − 6.62
			D_2233_ = 8.47 × 10^–10^	e_333_ = 23.24
			D_3333_ = 11.70 × 10^–10^	The piezoelectric constants without given values are all zero
		D_33_ = 1.43 × 10^–8^	D_1212_ = 2.35 × 10^–10^	
			D_1313_ = 2.30 × 10^–10^	
			D_2323_ = 2.30 × 10^–10^	

In this study, the material parameters of coal specimens were referred to as the characteristics of model materials in the destructive test of coal and rock conclusion by Chen^36^, including (1) The elastic modulus of coal was measured by WHY-type single-axis press. The elastic modulus of the coal seam was close to 5000 MPa by experiment, (2) The Poisson ratio of coal was 0.25 by the TAW-2000 microcomputer control rock servo triaxial pressure testing machine. The material parameters of coal are shown in Table 3.

**Table 3:** Material parameters of coal.

Specimen	Density (kg/m^3^)	Elastic modulus (Mpa)	Poisson ratio
Coal	1800	5000	0.25

The PZT patch adopts the C3D8E element type, which can simulate the piezoelectric material's piezoelectric effect, and the element's performance is relatively stable. The model of coal specimen adopts a C3D8R stress element (An 8-linear node brick, reduced integration, hourglass control). The solution result of this element for displacement is relatively accurate, and the accuracy of the analysis is not easily affected when the mesh is distorted.

#### Selection of excitation signal

**Figure 6:**
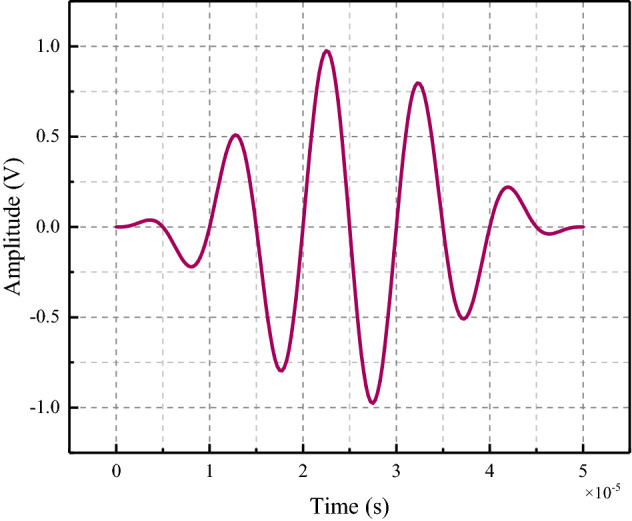
Excitation signal.

The excitation source used a 5-period 1 V five-peak wave modulated by the Hanning window, and the excitation signal is shown in Fig. 6. The potential of the electrode surface on the side where the coal surface and the PZT patch contact each other was set to 0. The excitation signal was input to the electrode surface on the side of PZT1 facing away from the coal surface and applied to this surface to simulate the emission and reception of stress waves. The excitation signal expressions are as follows^37^:

5 $$P(t) = \left\{ {\begin{array}{*{20}c} {0.5\left[ {1 - cos\left( {\frac{2\pi f}{N}t} \right)} \right]sin(2\pi ft),} & {\quad 0 \le t \le \frac{N}{f}} \\ {0,} & {\quad t > \frac{N}{f}} \\ \end{array} } \right.$$

where P(t) is the excitation signal, the excitation signal's center frequency, and N is the number of selected cycles. The excitation signal center frequency was 30 kHz and 100 kHz, and the periodicity N was set to 5.

#### Mesh

ABAQUS finite element software provides ABAQUS/Standard and ABAQUS/Explicit analysis modules. Among them, ABAQUS/Standard is a general analysis module for implicitly solving linear and nonlinear problems, which is suitable for dynamic, static, and complex nonlinear physical field coupling analysis problems. ABAQUS/Explicit is suitable for simulating transient and instantaneous dynamic ranges, such as explosion and shock. This simulation requires piezoelectric ceramics to generate and receive stress waves in the coal, which involves piezoelectric–electromechanical conversion analysis. However, the ABAQUS/Explicit module cannot realize the definition of the electrical boundary of piezoelectric ceramics. For the fine numerical simulation operation, the dynamic implicit analysis step in ABAQUS/Standard was selected for simulation calculation.

**Figure 7:**
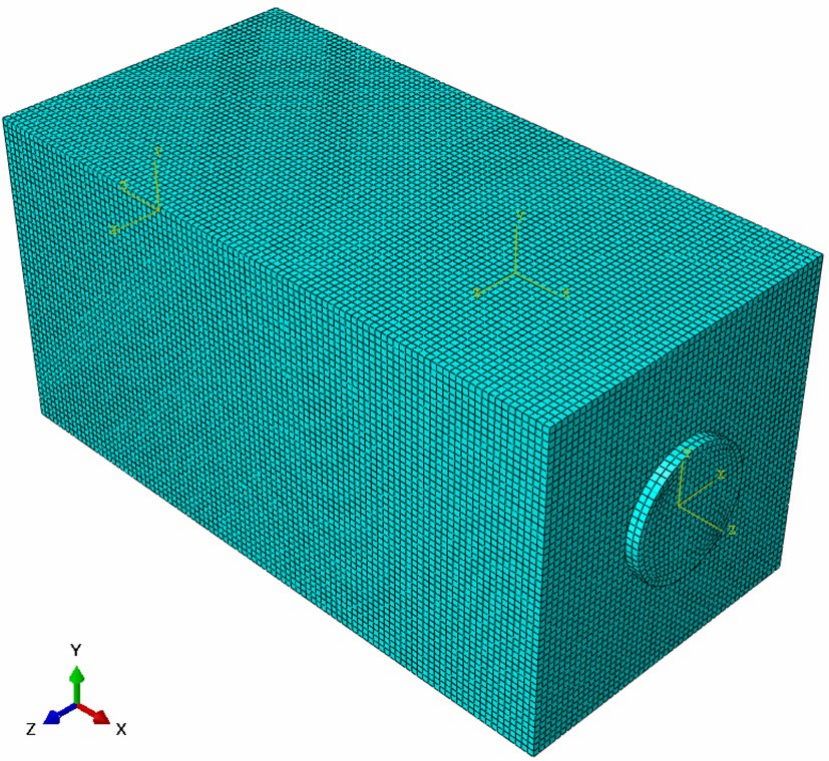
The schematic meshing of the model.

The accuracy of meshing was closely interrelated to the speed of model calculation and the accuracy of calculation results. In finite element simulation, if the mesh size is larger, the calculation time will be saved, but the accuracy of model calculation will be reduced. The fine mesh will improve the accuracy and make the calculation result closer to the actual result. Still, at the same time, it will lead to an increase in the calculation time, especially for complex models, which are more prominent. Therefore, to ensure the accuracy of the simulated stress wave propagation, the mesh size of the finite element must meet^38^:

6 $$l_{max} \le \frac{{\lambda_{min} }}{7}$$

where *l*_*max*_ is the maximum size of the mesh, *λ*_*min*_ is the wavelength of the minimum excitation signal. In this paper, the mesh size of the coal was set as 1 mm. The mesh division of the model is shown in Fig. 7.

## Results and discussion

### Results of experimental

The excitation signal in the experiment was a sine wave with frequency of 30 kHz and amplitude of 10 V. Because the power of the arbitrary function generator was small, in order to ensure that the sensor at the receiver can receive the transmitted signal completely, the signal was amplified by 10 times through the signal amplifier. The stress wave propagates in the specimen and finally reaches the PZT sensor. The electrical signal will be displayed to the oscilloscope, and the crack change information of the coal sample can be obtained by comparing the difference of the signals.

**Figure 8:**
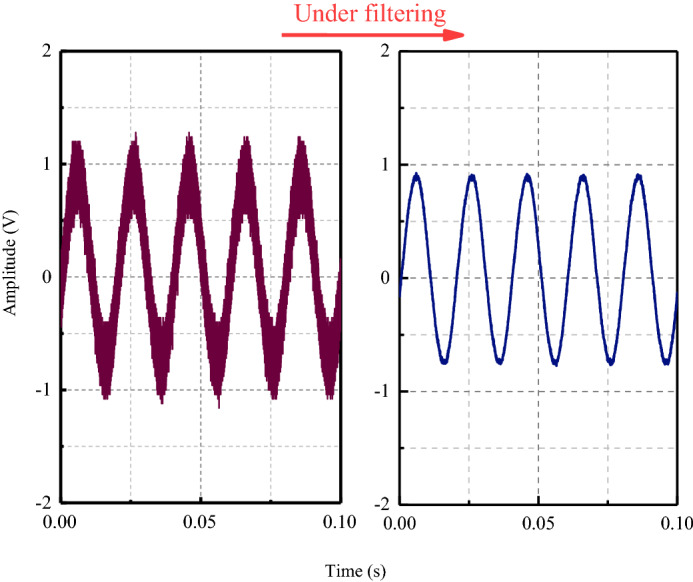
The waveform of a sinusoidal signal under filtering.

The MATLAB filtering program was used to filter the signal received by the PZT sensor, and the time domain waveform before and after the signal filtering was obtained, as shown in Fig. 8. (Take the signal without a crack as an example).

**Figure 9:**
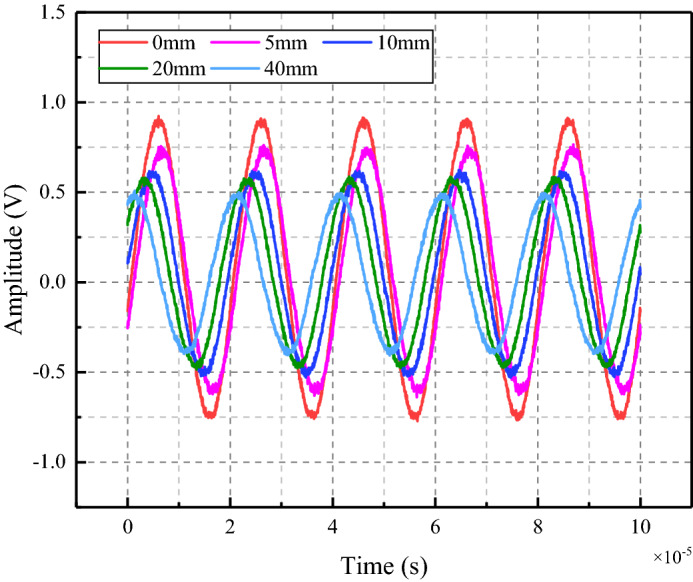
Time domain signal diagram received by PZT sensor.

According to the stress wave propagation theory, the piezoelectric actuator inputs the stress wave signal, and the piezoelectric sensor receives the stress wave signal. When the stress wave propagates in coal sample, it will carry the internal information of the coal sample. When there were cracks in the coal sample, the original propagation path of stress wave will be changed, which was reflected in the oscilloscope was the variation of the basic parameters such as the amplitude of the received signal. Therefore, the amplitude parameter analysis can be used as the basis for judging the fracture damage of coal sample. It can be seen from Fig. 9 that the appearance of cracks led to the rapid decline of the signal received by the PZT sensor. This was because the generation of cracks in coal sample the propagation of stress waves, resulting to the attenuation of the amplitude.

### Results of simulated

**Figure 10:**
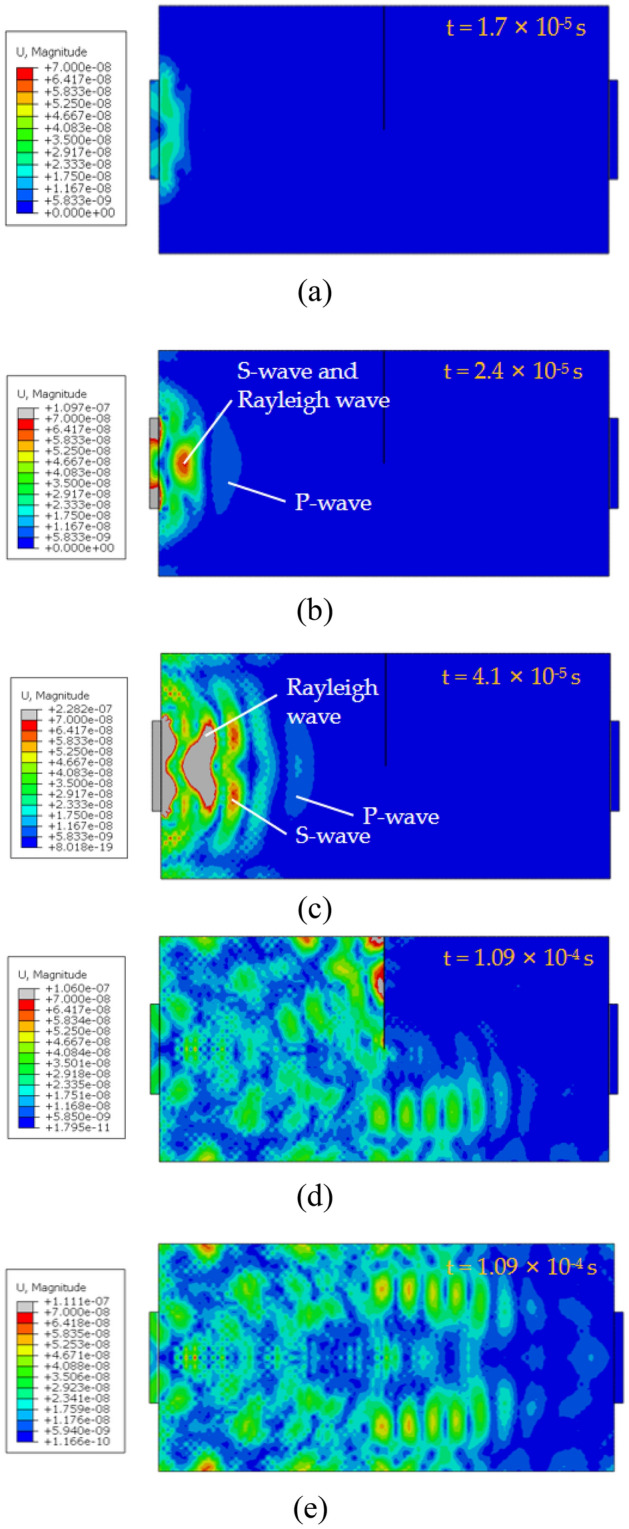
Snapshot of stress wave propagation.

The above model was simulated, and the wave field displacement diagram at different times was selected for analysis. According to the different propagation modes of elastic stress waves, they can be divided into body waves and surface waves. The body wave is centered at the excitation point and propagates in a spherical form from inside to outside in the medium, mainly including the S wave and P wave; Surface wave propagates outward along the surface of an elastic medium, mainly including Rayleigh wave (R-wave), Love wave and Stoneley wave. When the adjacent medium of the elastic medium is air or vacuum medium, and the thickness of the propagating medium is greater than the length of the surface wave, this kind of surface wave is called R-wave. As the propagation velocity of the S-wave, P-wave, and R-wave is different, they will be separated successively with time. PZT2 receives the stress waves of each component at different times. The propagation cloud diagram of the stress wave is shown in Fig. 10.

The sample with a coal gap depth of 25 mm was taken as an example. As shown in Fig. 10a, at the time t = 1.7 × 10^−5^ s, PZT1 exerts an excitation signal on the surface of the coal, under which stress waves of different components were generated. Because of the shorter running time, the stress waves of each component were not separated, and the types of stress waves cannot be distinguished. As shown in Fig. 10b, at the time t = 2.4 × 10^−5^ s, the p-wave propagates rapidly and gradually separates from the S-wave and R-wave, which were relatively backward and didn't separate. As shown in Fig. 10c, at the time t = 4.1 × 10^−5^ s, the P-wave, S-wave, and R-wave were separated, and the stress waves of each component can be distinguished. As shown in Fig. 10d, at t = 1.09 × 10^−4^ s, when the stress waves propagate to the crack, part of the wave is reflected and overlaps with the stress waves propagating to the right. Meanwhile, another part of the stress waves continues to propagate in Fig. 10d,e. When there are cracks and potholes in the structure, reflection or diffraction phenomena will occur in the process of stress wave propagation. The coal damage condition is recognized by comparing the change of the signal amplitude received by the PZT sensor at the condition of no crack and with crack.

### Data processing and analysis

Standard signal analysis methods usually include time domain analysis, frequency domain analysis, time difference domain analysis, and wavelet packet energy analysis. To find out the change law of signal under different conditions of coal crack, The two methods of time domain and frequency domain analysis were used to explore the variation law of the received signal under different depths of cracks and the evaluation index based on amplitude signal was defined.

#### Time domain analysis

**Figure 11:**
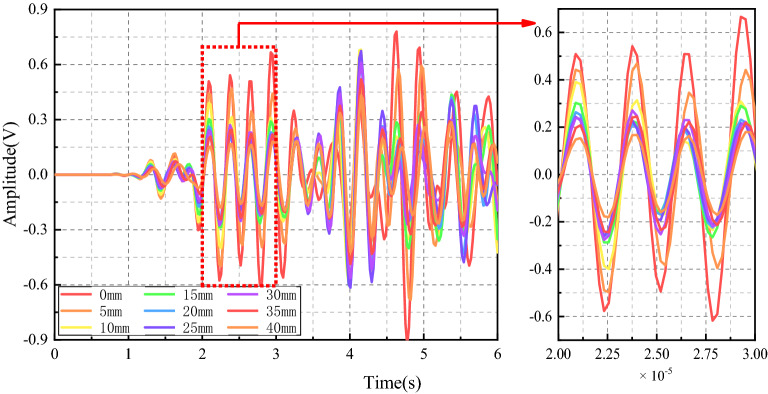
Time domain signal diagram received by PZT sensor (30 kHz).

**Figure 12:**
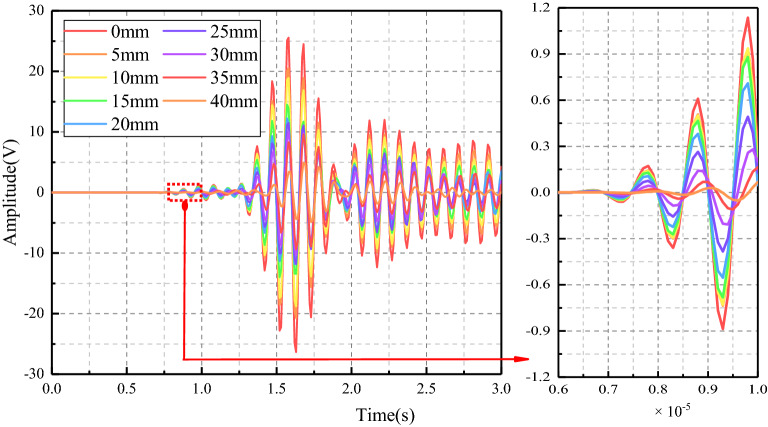
Time domain signal diagram received by PZT sensor (100 kHz).

In this paper, the maximum amplitude was chosen as the characteristic parameter to judge the crack depth of coal. The signal propagation of coal crack-free and crack depth of 5 mm, 10 mm, 15 mm, 20 mm, 25 mm, 30 mm, 35 mm, and 40 mm were simulated. The measured stress wave signal curves of the two excitation frequencies are shown in Figs. 11 and 12 respectively. When the excitation frequency is 30 kHz, which is consistent with the experiment, the curves of different crack depths showed obvious regularity only at the stage of 2 × 10^−5^ s to 3 × 10^−5^ s, while the curves in the remaining stages show irregular fluctuations. After the frequency is increased to 100 kHz, the whole curve has good regularity. Therefore, data with excitation frequency of 100 kHz is used for analysis in the simulation stage.

**Figure 13:**
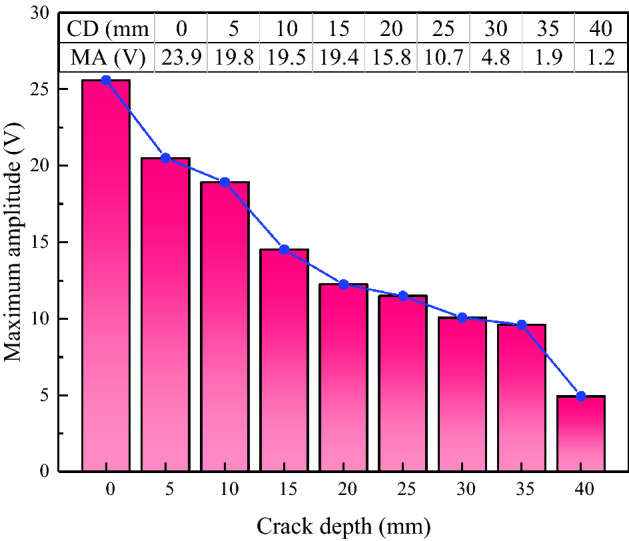
The maximum value of the signal at different crack depths.

It can be seen from Fig. 13 that the time domain signal of the coal specimen without crack was taken as the reference (in the figure, MA and CD are the abbreviations of Maximum amplitude and Crack depth, respectively.). By comparing it with coal in other conditions, it can be found that the time domain signal amplitude received was the largest by the specimen without crack. When there is a crack in the sample, the crack will inhibit the signal propagation and intensify the signal attenuation. The above results show that piezoelectric sensing activate technology can realize crack identification of coal structure.

#### Frequency domain analysis

**Figure 14:**
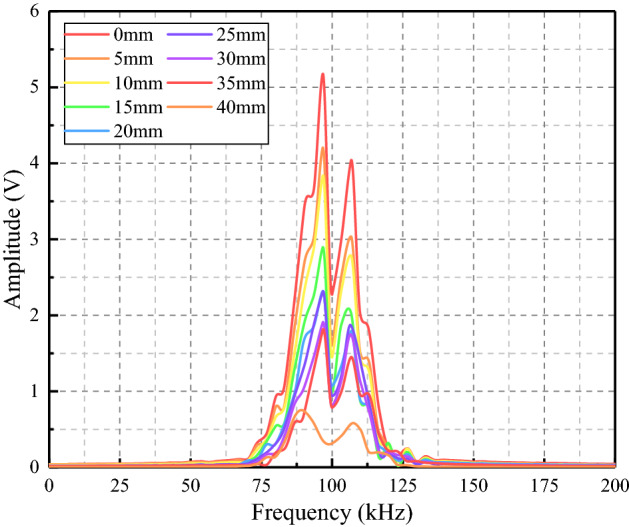
Frequency domain waveform.

In some cases, the fluctuation of the time domain signal was not apparent, and it was impossible to distinguish the signal's change visually. Therefore, we adopt the frequency domain analysis as a supplementary method. Frequency domain analysis is to transform time domain signals into frequency domain signals by Fourier transform for analysis. In this paper, the frequency domain waveforms at different crack depths were obtained after the Fourier transform of time domain signals by MATLAB, as shown in Fig. 14.

According to the figure, the peak amplitude of the spectrum pattern of the signal in each crack state corresponds to the same excitation frequency, indicating that the piezoelectric ceramic can accurately collect the corresponding excitation monitoring signal. With the increase of crack depth, the amplitude in frequency domain changes in the same way as that in time domain. It showed that when the monitoring signal frequency was the same, the signal waveform received by the PZT sensor in the frequency domain shrinks at different crack conditions.

**Figure 15:**
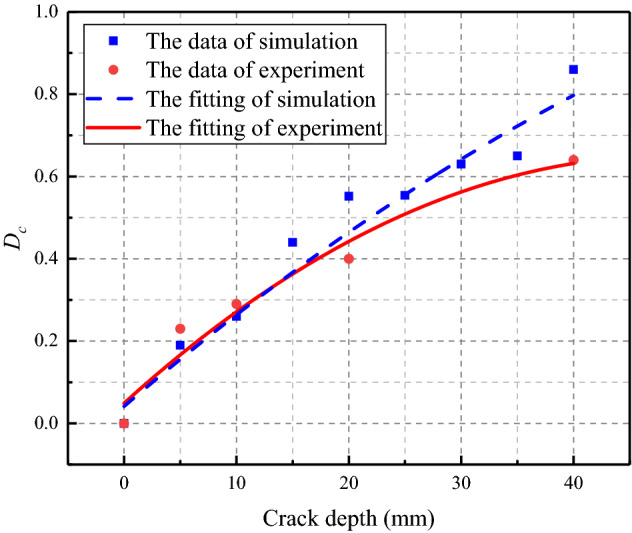
The degree of crack (${\mathrm{D}}_{\mathrm{c}}$).

To quantitatively evaluate the degree of cracking and to more intuitively see the difference between the signals received in the absence of cracks and those in the presence of cracks, In this paper, the evaluation index in degree of crack (*D*_*c*_) is defined based on the difference between the maximum values of frequency domain signals under different conditions. The formula is as follows:

7 $$D_{c} = \frac{{N_{max} - D_{max} }}{{N_{max} }}$$

where *D*_*max*_ is the maximum amplitude of the signal received by the sensor, *N*_*max*_ is the maximum amplitude of the signal received by the sensor at the condition of no crack, *D*_*c*_ is the index to measure the crack condition. The value range of *D*_*c*_ is between 0 and 1. The larger *D*_*c*_ is, the more serious the cracking degree of the crack is. The fitting curve of *D*_*c*_ calculated from the results of experimental and simulation are shown in Fig. 15. The value of *D*_*c*_ was 0 under the condition of no crack, and increases with the deepening of the coal sample crack. The existence and degree of crack can be well reflected by the value of *D*_*c*_. The above results have shown that the numerical simulation were consistent with the experimental results law. The fitted curves of experiment and simulation are in good agreement. The piezoelectric sensor technology can realize the identification of coal crack and verify the validity of numerical simulation.

## Conclusion

Taking the coal samples containing cracks and defects as the research object, this manuscript studied the propagation characteristics of stress waves in coal medium by means of theoretical analysis, numerical simulation and experimental verification, and proposed crack evaluation indexes, which provided experimental results, theoretical basis and scientific basis for coal health monitoring technology. The following conclusions can be made:

1. MATLAB filtered the extracted data, and temporal waveform before and after the signal filtering was obtained. The fracture depth perpendicular to the propagation direction of stress wave was negatively correlated with the signal amplitude, the deeper the crack, the smaller the amplitude.
2. To further explored the influence of coal structure crack on the signal frequency, the Fourier transform was used to transform the temporal waveform of the signal to the frequency domain waveform for frequency domain analysis. By comparing the frequency domain waveforms of the signals at different crack depths, it was found that the frequency domain signals were more sensitive to the crack changes.
3. The crack evaluation index *D*_*c*_ based on the maximum amplitude of the signal, and the value of *D*_*c*_ was 0 at the condition of no crack. When there was a crack in the coal, the crack depth was positively correlated with the *D*_*c*_, The deeper the crack, the larger the value of *D*_*c*_. The fluctuation method can be used for quantitative evaluation of the crack depth of coal structure and the *D*_*c*_ can well reflect the cracking condition of coal.

## Data Availability

The datasets generated during and analyzed during the current study are available from the corresponding author on reasonable request.
